# Non-specialized care of skin disorders: a cross-sectional survey of new patients attending dermatology clinic in a tertiary hospital in Jos, Northcentral Nigeria

**DOI:** 10.4314/ahs.v23i3.74

**Published:** 2023-09

**Authors:** Ruth O Adah, Collins John, Courage Uhunmwangho, Gabriel U Adah, Seline N Okolo

**Affiliations:** 1 Department of Paediatrics, Jos University Teaching Hospital Jos. Nigeria; 2 Department of Paediatrics, College of Health Sciences, University of Jos, Jos. Nigeria; 3 Department of Internal Medicine, College of Health Sciences, University of Jos, Jos. Nigeria; 4 National Primary Health Care Development Agency, Area 11.PMB 367, Garki, Abuja. Nigeria

**Keywords:** Non-specialized, skin diseases, unorthodox, treatment, dermatology, clinic

## Abstract

**Background:**

Given the paucity of skin health specialists in Nigeria and the low level of awareness amongst its populace, patients seek for care for skin related disorders from different sources and are given a variety of remedies before accessing specialist care.

**Objectives:**

This study was aimed at describing outlets visited and medication received by patients with skin disorders prior to attending the dermatology outpatient clinic in JUTH.

**Methods:**

This was a cross sectional study conducted over one year. Information on socio-demography, sources and medication received prior to presentation was obtained and analysed using SPSS 23.

**Results:**

The male: female ratio among 166 consenting new patients was 1:1.4. Prior to presentation patients sought care most frequently from Health facilities (68.1%), Patent medicine vendors-PMV (30.7%) and Traditional healers (21.7%). Overall, different steroid preparations were the most commonly used medications (56.6%) across all age groups with fixed combination preparations most frequently used (32.5%). Unconventional substances reportedly used by patients for skin disorders were urine, toothpaste, tomatoes, salt, water in which a life catfish had been kept and fats from a dead dog. Only 21.1% of the patients did not use any medication prior to presentation.

**Conclusion:**

There is need to increase capacity in the care of common skin diseases at all levels of the health care system to decrease patronage of unconventional providers. Raising awareness of the general public on the potential dangers of inappropriate treatment of skin diseases and strengthen referral system is imperative to reduce the burden of skin diseases in the country.

## Introduction

Skin problems are among the most common diseases affecting different age groups world- wide.^1^ It has been reported to be one of the commonest reasons to access primary health care with a new health problem.^2^ The financial implications of treating skin diseases with the specific morbidity from the discomfort, disfigurement, disability contributes to a reduction in quality of life.^1^, ^3^

In Africa, the burden of skin diseases is high in its most populous nation- Nigeria with prevalence rates documented in school and community studies ranging from 19.2% to 64.2% and accounting for as much as a third of outpatient medical consultations.^4^-^9^ Accessing dermatology services within, Nigeria is not as easy as in the developed nations where dermatologists or the General Practitioners(GP) with capacity to manage common skin disorders are usually the first point of contact.^2^,^9^ The paucity of skin health specialist, poor capacity by general practitioners to manage skin disorders, high level of ignorance and poverty among the populace are reasons patients ‘meander’ through various orthodox and non-orthodox health facilities seeking dermatologic care.^9^-^11^ Individuals who have skin disorders like other morbidities in developing nations tend to have used a variety of medications. A large proportion of persons suffering from skin diseases often access treatment from Patent Medicine Vendors (PMV) found in both rural and urban communities. There they are offered a variety of oral or topical medication and even inappropriate procedures such as surgical excisions carried out on them.^11^-^13^ The recurrent or persistent nature of some skin diseases have led some to seek religious/spiritual help. Traditional healers use known beneficial substances, and even some with unknown benefits. These together may increase the risk for local and systemic complications before accessing specialist care.^5^,^12^,^13^,^15^,^16^ While these challenges add to the burden of skin diseases on individual and families, they however have not been viewed as significant enough to warrant public health interventions in the country.^17^ This study was aimed at describing sociodemographic characteristics of new patients attending the dermatology outpatient clinic in JUTH. Also, to document their experiences as it relates to places visited for treatment and medications used prior to attending the clinic

The study was a descriptive cross-sectional one conducted in the Paediatric dermatology outpatient clinic in the Jos University Teaching Hospital (JUTH), Plateau State. JUTH is a referral centre for the North-central region of Nigeria but receiving referrals from beyond, mainly from 5 surrounding Northern States (Gombe, Nasarawa, Benue, Bauchi and kogi states). The Paediatric Dermatology Outpatient Clinic was started in the year 2012, specifically to attend to children with skin related morbidity. However due to the scarcity of dermatologist in Jos and the North-central Nigerian region, the clinic has recently been open to patients above 18 years of age. It is run as a weekly clinic for referred patients from within and outside the state. An average of about 8 new patients are seen weekly in addition to those on follow up.

A semi structured questionnaire was administered on all consenting new patients attending the dermatology clinic for the first time during the study period (June 2019-May 2020). The patients were recruited consecutively and the questionnaire was used to obtain Information on the socio-demographic characteristics of the patients, places where treatment was sought and treatment received prior to presentation in the dermatology clinic. Patients volunteered the names of the drugs used for their skin conditions or brought the empty packs or drugs themselves for identification and classification. Medication used that could not be recalled or produced were classified as unknown. Subsequently, diagnosis was obtained after examination and investigations. The data obtained was analysed using SPSS (Statistical Package for Social Sciences, Chicago, USA) version 23. Frequency tables were used to summarize the categorical variables, the association between patient characteristics and the sources and types of treatment received was tested using chi-square test with a Probability value of less than 0.05 considered as significant.

## Results

### Types of skin diseases observed in new patients attending the skin clinic

There were 174 skin diseases seen in the new patients as some had multiple conditions. They were put into 7 main groups- the group called ‘Others’ consisted of disease entities with fewer than 4 patients in those the category, such as; Pruritic Papular Eruptions of HIV, keratosis pilaris, benign tumours, keloids, Corns and calluses.

### Sociodemographic characteristics of subjects

A total of 166 new patients representing 40.3% of the 412 patients were seen in the dermatology clinic over a period of one year. A one-sample-chi square goodness of fit was used to test the differences in sociodemographic characteristic of subjects of the study. The age groups differed significantly(p-0.000) from each other with children of school age forming the largest age group (32.5%). Females formed a significantly higher portion of new patients attending the clinic (57.8%; p-0.44) with a Male: Female ratio of 1: 1.4. Only 15.7% of the patients were on health insurance (National Health Insurance Scheme-NHIS which was significantly less than those who paid out of pocket(p-0.000)

### Avenues of treatment prior to attending dermatology clinic

While about a fifth (19.9 %) came directly to JUTH for specialty care. The place frequently visited by patients to access care for skin conditions, prior to presentation were health facilities (68.1%) with 38% visiting HF exclusively. Patent medicine Vendors- PMV (30.7%) and traditional healers (21.7%).

### Medication used by new patients prior to attending the dermatology clinic

Only about a fifth (21.1%) of the new patients hadn't taken any medication before presenting at the clinic. With more than half (56.6%) having used a form of steroid or more. The combination creams (containing antibiotics, antifungals and steroids) were the commonest preparation used (32.5%) of all medications. About a quarter (24.7%) used traditional medication for their skin lesions, while some used bizarre substances to treat skin disorders.

### Relationship between age of patient and place visited for treatment prior to presentation

Infants had the lowest proportion of those that visited non-orthodox facilities, multiple outlets and used any form of medication, while teenagers had the highest percentage of use of combination cream (61.5%, p-0.049) as well as steroids (69.2%). Patients ≥18years had significantly higher proportions of those who used, traditional medication, antibiotics, antifungals and sought care in 2 or more places, compared to all other age groups.

### Relationship between places visited prior to presentation, medication used and sex of patients

While a higher proportion of males (81.4%vs77.1%) received treatment in general prior to presentation, a significantly greater proportion of females received steroids as medications prior to presenting in the dermatology clinic (64.6% vs 45.7%, p-0.015)

### Relationship between places visited prior to presentation with medication used and mode of payment

A greater percentage of persons who paid out of pocket when compared to those on NHIS visited non-orthodox outlets and multiple places, used some medication prior to presentation although the differences were not statically significant. Less than half the proportion of patients on health insurance in comparison to those who pay out of pocket had used combination creams (15.4% vs 35.7%; p-0.042)

## Discussion

Our outpatient dermatology clinic with a relatively small patient load when compared to other reports still has a substantial proportion of new patients in comparison to total patients. This pattern is also noted in other dermatology clinics and is a reflection of the burden of skin diseases in the community.^18^,^19^ The prevalence of skin disorders has been shown to be high in Africa and constitute a significant proportion of outpatient visits to health facilities so it is not remarkable that new patients form a significant portion in our clinic.^1^,^6^

The higher number of females compared to males among new patients attending dermatology clinics has also been noted in previous studies in the country.^17^,^7^,^20^ The bulk of the patients being in the pre-school and school age (1-12years) may be attributed to the wide range of skin diseases that are prevalent in this age group; by this age, most children would have experienced one or more skin related morbidity.^19^

Dermatitis was the commonest disease found in new patients with a rate of 32.2%, almost doubling the rate of infections and infestations at 18.4%. Although not a common finding in Africa in the past, it has been observed in a few other studies.^7^,^20^,^21^ This is in contrast to a previous report from a community-based study among school aged children in same city of Jos showing infections as the commonest skin disorder, doubling the prevalence rate of dermatitis at that time.^8^ Although, differences between community and hospital-based studies may explain this variance, it could also mean infections are being managed in the community and at the other levels of healthcare reducing the need for referral to the tertiary centre for specialist care.^1^ The pattern differs from those described in many hospital-based studies across Africa where infections have been found as prominent.^6^,^17^,^22^ Dermatitis and infections account for a significant subset of dermatologic conditions in the under-five and school age group that form the bulk of our first time subjects.^23^,^24^ It could explain the difference between our findings and these previous studies. Other likely reasons are the differences in sample size, design and geography.

The small proportion of new patients (38.0%) that visited health facilities alone and even smaller proportion (19.9%) that presented directly to our centre for health care prior to presenting in the specialty clinic represent the minority, with the appropriate approach in seeking care for dermatological conditions. And for this reason, primary health care providers ought to promote correct health seeking behaviour, have basic skills to intervene early in cases of skin disorder and refer appropriately.

With the overwhelming majority of the patients paying out of pocket in JUTH which is an NHIS designated hospital and the only centre rendering specialty dermatological services within the state, the issues of geographical and financial access to quality skin health care is brought to the fore. Our study found that subjects with health insurance cover tended towards seeking care in Health facilities only and sought healthcare in fewer centers prior to attending the specialty clinic than those who paid out of pocket. They were less likely to have used a variety of medications (steroids, combination creams, antibiotics and traditional medication). This agrees with several reports showing better health seeking behaviour and higher utilization of health care services by individuals with health insurance; by extension, this has the potential to promote appropriate health seeking behaviour as it concerns dermatology patients.^25^,^26^ The management of skin disorders like other diseases often needs repeat or follow-up visitations; thus fees for service, drugs, investigations, transportation contribute to the burden of skin disorders on individuals and families.^11^,^24^ The impoverishing effect of out-of-pocket expenditure is well known, so measures to improve access to quality skin health care through health insurance ought to be explored as a step towards expanding health coverage.^27^

The significant proportion of subjects that sought unorthodox services is similar to that of Yusuf et al in kano, another city in the northern region of Nigeria who showed substantial proportion of patients preferred accessing similar non-orthodox services prior to accessing specialty care for reasons of cost and non-availability of dermatological services.^11^

It is of concern to note that while antifungals and antibiotics were used by significantly higher proportions of patients older than 18 years, steroids had been used by the majority (56.6%) of patients across all age groups. It is interesting to note that in our study, care givers who reported not administering any antifungal, antibiotics and traditional mediation to subjects less than one year-old had used steroids for these same infants suggesting, substantial use of steroids in the younger age groups, although the sample size of subjects in that age group was too small to draw meaningful conclusions. On the other hand, there seem to be some health seeking behaviour that protects infants as significantly less were taken to multiple avenues or given other medication prior to attending the specialty clinic. It could be explained by the probable awareness of infants' vulnerability and care in avoiding local and systemic complications of steroids, traditional and unknown medication by caregivers,’ community members and health workers.^28^ Similar sensitivities could be employed to emphasis cautious use of steroids in infants among caregivers and health care providers. Along with the predominant use of steroids by females, these findings are in consonance with those from a study in south east Nigeria, where 58.7% of new patients were found to have used topical steroids mainly for cosmetic purposes.^7^

The fixed drug combination creams which were the commonest steroids preparations used by a considerable percentage of teenagers has been prohibited in children and banned by the Indian Government and the US Food and Drug Administration (FDA).^29^,^30^ It is thus essential that regulatory measures are put in place in response to its widespread availability and use in Nigeria as suggested by Ibekwe *et al.*^13^

Furthermore, the extent that a few individuals had gone to, by using bizarre substances such as one owns urine may depict level of ignorance. anxiety or desperation that may be associated with skin disorders. Many of these unconventional treatments exposes patients to potential harm. Raising awareness of the general public on the potential dangers of inappropriate treatment of skin diseases and places to visit for skin related health care is imperative to reduce the burden of skin diseases in the country. This study had 2 main limitations –a significant proportion of patients did not know or could not recall medications previously used. Also answers given may tend towards social desirability so accurate narratives of places visited and medication used may not be fully divulged.

## Conclusion

A significant proportion of them sought care for skin disorders in multiple places, health facilities, patent medicine shops and traditional healing centres were the commonest among a wide range of places visited for dermatological care. The bulk of first-time patients paid out of pocket for services. The fixed combination topical steroid creams were the most frequently used medications prior to attending the dermatology clinic. This study highlights the need for education of the public on appropriate health care seeking behaviour as it concerns skin disease along with instituting a sound referral system to ease access. More information is required on the effects of these commonly used topical steroids on the Nigerian child and possibility of their control.

## Figures and Tables

**Table I:** Types of skin diseases observed in new patients attending the skin clinic

Skin disease groups	Cases (number)	Percentage %
Dermatitis	56	32.2
Infections and infestations	32	18.4
Papulosquamous disorders	19	10.9
Urticaria and angioedema	15	8.6
Disorders of pigmentation	10	5.7
Pilosebaceous	9	5.2
Others	33	19
Total	174	100

**Table II:** Sociodemographic Characteristics of subjects

Variables	Number (percentage)	X2	p
**Age Group (years)**			
<1	10(6.0)	55.867	0.000*
1-4	55(33.1)		
5-12	54(32.5)		
13-18	13(7.8)		
≥ 18	34(20.5)		
Total	166(100)		

**Sex**			
Male	70(42.2)	4.072	0.044*
Female	96(57.8)		
Total	166(100)		
**Mode of payment**			
On health Insurance	26(15.7)	78.289	0.000*
Out of pocket payment	140(84.3)		
Total	166(100)		

• Statistically significant

**Table III:** Avenues of treatment prior to attending dermatology clinic

Avenues of treatment prior to attending dermatology clinic	Number who accessed treatment avenue #	Percentage
Presented directly to JUTH only.	33	19.9
Attended other health facilities.	80	48.2
Patent medicine vendors.	51	30.7
Traditional healers.	36	21.7
Commercial laboratories.	26	15.7
Relatives/friends.	21	12.7
Health Worker outside facility.	10	5.7
Beauticians /cosmetician.	7	4.2
Faith based healers.	7	4.2

*# Treatment avenues not mutually exclusive of each other; thus, total don't add up to 166*.		

**Facility visited as exclusive groups(n=166)**		
Visited Health Facility only.	63	38.0
Visited Non- orthodox outlets only (non health facilities).	57	34.3
Visited both orthodox Health Facility and Non-orthodox outlets.	46	27.7
Total	166	100

**Table IV:** Medication used by new patients prior to attending the dermatology clinic

Medication received prior to attending specialty clinic	Number of patients who used each medicine type #	Percentage (%)
Didn't take any medication prior to presentation	35	20.1
Any Steroid based preparations	94	56.6
- Triple combination creams	54	32.5
- Plain topical steroids creams	46	27.7
- Oral steroids	17	10.2
Any Antifungal preparation	35	21.1
- Topical antifungals	35	21.1
- Oral antifungal	22	13.3
Antibiotics (oral)	36	21.7
Antihistamines (oral)	21	12.7
Traditional medication (oral and topical)	41	24.7
Unknown medication (topical and orals)	23	13.9
Others (urine, toothpaste, tomatoes, salt, water in which catfish was soaked and fats from a killed dog)	**6**	**3.6**

# Responders may mention multiple medications used which are not mutually exclusive; thus, total don't add up to 166.

**Table V:** Relationship between Age of patient and place visited for treatment prior to presentation

Places visited prior to presentation	<1year (n=10)	1-4years (n=55)	5-12years (n=54)	13-17 years (n=13)	≥18years (n=34)	X^2^	*P*
Health Facility only	6(60.0)	21(38.2))	20 (37.0)	5(38.5)	11(32.4)	6.811	0.557
Non-orthodox outlets	2(20.0)	20(36.4)	20(37.0))	6(46.2)	9(26.5)		
Combined	2(20.0)	14(25.5)	14(25.9)	2(15.4)	14(41.2)		
Total	10(100)	55(100)	54(100)	13(100)	34(100)		

**Number of places visited prior to presentation**							
0 place	7(70.0)	8(14.5))	12(22.2)	2(15.4)	4(11.8)	24.227	0.002*
1 place	2(20.0)	29(52.7)	23(42.6)	7(53.8)	11(32.4)		
2 and above places	1(10.0)	18(32.7)	19(35.2)	4(30.8)	19(55.0)		
Total	10(100)	55(100)	54(100)	13(100)	34(100)		

**Received any form of treatment prior to presentation**							
Yes	4(40)	49(89.1)	40(74.1)	10(76.9)	28(82.4)	13.557	0.009*
No	6(60)	6(10.9)	14(25.9)	3(23.1)	6(17.6)		
Total	10(100)	55(100)	54(100)	13(100)	34(100)		

**Used steroid prior to presentation**							
Yes	3(30.0)	34(61.8)	25(46.3)	9(69.2)	23(67.6)	8.359	0.079
No	7(70.0)	21(38.2)	29(53.7)	4(30.8)	11(32.4)		
total	10(100)	55(100)	54(100)	8(100)	34(100)		
**Used combination creams prior to presentation**							
Yes	1(10.0)	20(36.4)	13(24.1)	8(61.5)	12(35.3)	9.543	0.049*
No	9 (90)	35(63.6)	41(75.9)	5(38.5)	22(64.7)		
Total	10(100)	55(100)	54(100)	8(100)	34(100)		
**Used antifungal**							
prior							
Yes	0(0)	9(16.4)	10(18.5)	3(23.1)	13(38.2)	9.664	0.046*
No	10(100)	46(83.6)	44(81.5)	10(76.9)	21(61.8)		
Total	10(100)	55(100)	54(100)	13(100)	34(100)		
**Used Antibiotics**							
Yes	0(0.0)	9(16.4)	10(18.5)	3(23.1)	13(38.2)	10.487	0.033*
No	10(100)	46(83.6)	44(81.5)	10(76.9)	21(61.8)		
Total	10(100)	55(100)	54(100)	13(100)	34(100)		
**Used Traditional medication**							
Yes	1(10)	17(30.9)	17(31.5)	2(15.2)	15(44.1)	6.242	0.182
No	9(90)	38(69.1)	37(68.5)	11(84.6)	19 (55.9)		
Total	10(100)	55(100)	54(100)	13(100)	34(100)		

* Statistically significant

**Table VI:** Relationship between places visited prior to presentation, medication used and sex of patients

Places visited prior to presentation	Male (n-70)	Female(n-96)	X2	P
Health facility only	27 (38.6)	36(37.5)	0.253	0.881
Non-orthodox outlets	25(35.7)	32(33.3)		
Combined	18(25.7)	28(29.2)		
Total	70 (100)	96(100)		

**Number of places visited prior to presentation**				
0 place	14(20.0)	19(19.8)	0.896	0.639
1 place	33 (47.1)	39(40.6)		
2 or more places	23(32.9)	38(39.6)		
Total	70(100)	96(100)		

**Received any form of treatment prior to presentation**				
Yes	57(81.4)	74(77.1)	0.459	0.498
No	13(18.6)	22(22.9)		
Total	70(100)	96(100)		

**Use of steroid prior to presentation**				
Yes	32(45.7)	62(64.6)	5.868	0.015*
No	38(54.3)	34(35.4)		
Total	70(100)	96(100)		

**Use of combination creams prior to presentation**				
Yes	19(27.1)	35(36.5)	1.601	0.206
No	51(72.9)	61(63.5)		
Total	70(100)	96(100)		
**Use of any antifungal prior**				
Yes	14(20.0)	21(21.9)	0.086	0.770
No	56(80)	75(78.1)		
Total	70(100)	96(100)		

**Use of antibiotics prior to presentation**				
Yes	14(20.0)	22(22.9)	0.203	0.652
No	56(80.0)	74(77.1)		
Total	70(100)	96(100)		

**Use of traditional medicine prior to presentation**				
Yes	23(32.9)	29(30.2)	0.132	0.716
No	47(67.1)	67(69.8)		
Total	70 (100)	96(100)		

* Statistically significant

**Table VII:** Relationship between places visited prior to presentation with medication used and mode of payment

Places visited prior to presentation	Out of pocket (n-140)	On health insurance (n-26)	X2	P
Health Facility only	49 (35.0)	14(53.8)	3.429	0.180
Non-orthodox outlets	51(36.4)	6(23.1)		
Combined	40(28.6)	6(23.1)		
Total	140 (100)	26(100)		

**Number of places visited prior to presentation**				
0 place	26(18.6)	7(26.9)	0.979	0.613
1 place	62 (44.3)	10(38.5)		
2 or more places	52(37.1)	9(34.6)		
Total	140(100)	26(100)		

**Received any form of treatment prior to presentation**				
Yes	110(78.6)	21(80.8)	0.64	0.801
No	30(21.4)	5 (19.2)		
Total	140(100)	26(100)		

**Use of steroid prior to presentation**				
Yes	82(58.6)	12(46.2)	1.377	0.241
No	58(41.4)	14(53.8)		
Total	140(100)	26(100)		

**Use of combination creams prior to presentation**				
Yes	50(35.7)	4(15.4)	4.129	0.042*
No	90(64.3)	22(84.6)		
Total	140(100)	26(100)		

**Use of any antifungal prior**				
Yes	29(20.7)	6(23.1)	0.74	0.786
No	111(79.3)	20(76.9)		
Total	140(100)	26(100)		

**Use of antibiotics prior to presentation**				
**Yes**	32(22.9)	4(15.4)	0.721	0.396
**No**	108(77.1)	22(84.6)		
**Total**	140(100)	26(100)		

**Use of traditional medicine prior to presentation**				
Yes	37(26.4)	4(15.4)	1.438	0.230
No	103(67.1)	22(84.6)		
Total	140 (100)	26(100)		

* Statistically significant
